# Enhanced Light Sheet Elastic Scattering Microscopy by Using a Supercontinuum Laser

**DOI:** 10.3390/mps2030057

**Published:** 2019-07-05

**Authors:** Diego Di Battista, David Merino, Giannis Zacharakis, Pablo Loza-Alvarez, Omar E. Olarte

**Affiliations:** 1 Institute of Electronic Structure and Laser, Foundation for Research and Technology-Hellas, N. Plastira 100, 70013 Heraklion, Greece; 2 Materials Science and Technology Department, University of Crete, 71003 Heraklion, Greece; 3 UOC-Universitat Oberta de Catalunya, Rambla Poblenou 156, 08018 Barcelona, Spain; 4 ICFO-Institut de Ciencies Fotoniques, The Barcelona Institute of Science and Technology, 08860 Castelldefels, Spain; 5 Vicerrectoría de Investigación, Universidad ECCI, 111311 Bogotá, Colombia

**Keywords:** Light Sheet Fluorescence Microscopy, biological microscopy, laser speckle, elastic scattering

## Abstract

Light sheet fluorescence microscopy techniques have revolutionized biological microscopy enabling low-phototoxic long-term 3D imaging of living samples. Although there exist many light sheet microscopy (LSM) implementations relying on fluorescence, just a few works have paid attention to the laser elastic scattering source of contrast available in every light sheet microscope. Interestingly, elastic scattering can potentially disclose valuable information from the structure and composition of the sample at different spatial scales. However, when coherent scattered light is detected with a camera sensor, a speckled intensity is generated on top of the native imaged features, compromising their visibility. In this work, we propose a novel light sheet based optical setup which implements three strategies for dealing with speckles of elastic scattering images: (i) polarization filtering; (ii) reducing the temporal coherence of the excitation laser light; and, (iii) reducing the spatial coherence of the light sheet. Finally, we show how these strategies enable pristine light-sheet elastic-scattering imaging of structural features in challenging biological samples avoiding the deleterious effects of speckle, and without relying on, but complementing, fluorescent labelling.

## 1. Introduction

Light sheet fluorescence microscopy (LSFM) is a simple but powerful imaging technique which enables fast and non-phototoxic 3D inspection of living specimens [1,2]. Most LSFM implementations rely on sample labelling with fluorescent reporters for imaging the targeted biological structures. This provides the specificity advantage of fluorescence microscopes that makes them successful in all sorts of biological experiments. A laser light sheet that passes through the sample mediates fluorescence excitation in LSFM. During its propagation through the tissue, part of the laser light scatters towards the detection camera, light that is usually blocked and discarded by using a specific filter [2]. However, this scattered light could carry valuable information about the structure and composition of the sample. When the scattering is inelastic, for example Raman scattering, the obtained spectrum can provide information on the chemical composition of the samples in 3D [3]. On the other hand, when the scattering is elastic, it discloses information on the structure of the sample at different spatial scales: much smaller than (Rayleigh scattering), comparable to (Mie scattering), or even much larger (Geometric scattering) than the wavelength of light. This is due to the fact that changes in the refractive index, the sources of light scattering, are a measure of local molecular density and, as such, of the biological sample architecture [4,5]. Elastic scattering from the samples is rarely used as a source of contrast for biological imaging, with the exception of optical coherence tomography (OCT) techniques. OCT relies on the infrared light backscattered by the sample to produce cross-sectional images of tissues [6]. In terms of resolution and penetration depth, OCT stands between ultrasound imaging and optical microscopy, and, due to its versatility has become an important tool in many areas of medicine [7]. Nevertheless, when the elastic scattering of coherent light is used for OCT, or other imaging modalities, a complex interference field is generated upon propagation through the sample due to the heterogeneous refractive index typical of tissues and other cell complexes. This field is known as the “speckle pattern” due to its grainy appearance and, for imaging applications, it is usually considered detrimental as it adds up to the features of interest [8]. In some applications, and when wavefront shaping is applied, the speckle pattern can be exploited to overcome scattering and diffusion in opaque samples, but not without limitations in terms of complexity and general applicability [9,10].

Therefore, speckle makes elastic scattering a bad candidate as a source of contrast for light-sheet imaging as it introduces undesired local intensity modulations that are quite undistinguishable from the intensity contrast generated by a sample’s own features. In spite of this, light sheet elastic scattering microscopy has been already implemented for plant root phenotyping, where the image quality was shown to be dependent on the turbidity of both the mounting substrate and the sample [11]. For diminishing a substrate’s induced background and speckle noise, this technique was further updated to include polarization filtering and axial scanning, at the cost of reducing the axial resolution of the microscope [12]. Another different implementation based on elastic scattering uses a low-coherence light sheet illumination, instead of the axial scanning, for speckle reduction. In this case, a stack of coverslips was utilized to generate a set of light sheets propagating at different angles which are summed together to cancel out the speckles [13]. This technique was applied to enhance the visualization of unstained morphologic neuronal structures of mice brains.

In this work, despite the speckle formation, we show that by using not only polarization filtering but also by allowing the full control of the light-sheet coherence, it is possible to retrieve the sample’s intrinsic contrast and the image resolution from the elastic-scattered light.

First, to have control over temporal coherence we use a supercontinuum fibre laser (SCL) which delivers an ultra-broadband white-light spectra with a single mode beam [14,15]. These light sources, due to their large emission bandwidth, present low temporal coherence and, therefore, can help to reduce the contrast of the speckles when used for imaging applications [8,16]. In addition, the emission of an SCL occurs through a single mode optical fibre, generating a beam that can be further focused to a diffraction-limited spot, achieving both the resolution and the intensities required for microscopy applications. Due to their extended emission wavelength range, spanning from the visible to the infrared, SCLs are becoming popular as compact light sources for confocal microscopes and other emerging optical techniques, such as the enhanced-resolution visible light OCT [17].

Second, to reduce the spatial coherence of the elastic-scattering light-sheet images, we introduce angular diversity at the illuminated plane of the sample. For this, instead of keeping the light sheet static during the recording time of the camera, we use a pivoting light-sheet scanning approach. This is similar to the method used in the previous reports for avoiding “shadows” in LSFM [18,19]. In this case, the speckle related to the spatial coherence of the laser beam can be reduced by creating angular and spatial diversity as the light sheet propagates though different paths across the substrate and the sample. Here, uncorrelated speckle patterns are generated upon scanning for the different angular states of the light sheet. Such speckle realizations are averaged out during the integration time of the camera. In this sense, the beam scanning frequency determines the amount of speckle reduction: if the number of speckle realizations per single frame is increased, less visibility of the speckles is expected.

Finally, we show that, by using our polarization filtered low-coherence light sheet imaging approach, both the substrate background and the speckle noise can be highly reduced in testing samples. Furthermore, we have tested our enhanced elastic scattering light sheet microscope in combination with LSFM by imaging challenging and biologically relevant samples. In particular, we show that the volumetric morphological information obtained using elastic scattering perfectly complements standard fluorescent imaging from labelled structures.

## 2. Experimental Setup

**Figure 1:**
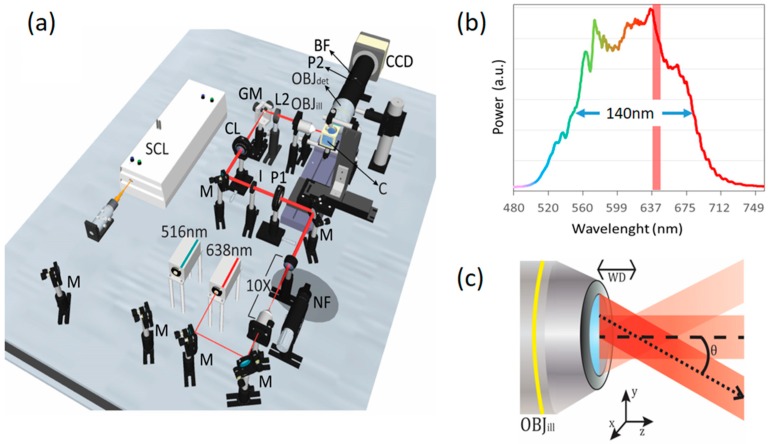
Scheme of the experimental setup for polarization and coherence control in elastic scattering light sheet microscopy. In (**a**) the experimental setup implemented is shown. The light sheet illumination path consists of a couple of diode lasers emitting at 515 nm and 638 nm, and a super-continuum laser (SCL). Laser beams are expanded 10 times before entering the microscope. P1 is a half-wave plate (HWP) which controls the polarization of the three beams before passing through the cylindrical lens (CL), the galvo mirror (GM) and the illumination objective (OBJill). GM scans the beam at OBJill’s pupil generating a pivoting light sheet at the sample plane. Samples are kept within a custom-made immersion chamber (C) filled with water. The detection system is composed of a 0.5 N.A. objective lens (OBJdet), a 200 mm tube lens (for a total magnification of 20X) and polarizer (P2). (**b**) The emission spectrum of the SCL in the band 500–700 nm (140 nm FWHM), compared to the bandwidth (1.2 nm) of the red diode laser (Red vertical band). (**c**) Detail of the optical setup close to the illumination objective lens (OBJill), illustrating the pivoting light sheet approach. The laser light sheet pivots around an axis located at the working distance (WD) of OBJill, i.e., at the centre of the sample plane. See the main text for more details.

A schematic of our optical setup is depicted in Figure 1a. The main component of our elastic scattering light sheet microscope is a supercontinuum fiber laser (FYLA SCT500, Paterna, Spain) which emits a broadband spectrum of light from the visible to the infrared. For this work, we have selected a band from 500 to 700 nm (140 nm FWHM), hence offering a lower temporal coherence for reduced speckle contrast. In addition, for comparison purposes, we have included other two high-coherence lasers to our setup, emitting at 516 nm (Toptica iBeam smart, 10 nm bandwidth) and 638 nm (Cobolt 06-01 MLD, bandwidth < 1.2 nm FWHM), respectively. The bandwidth selected for the SCL operation was chosen to: (i) match the quantum efficiency profile of the charge-coupled device (CCD) camera (>50% in the range 400–700 nm); (ii) lie in a wavelength range where the SCL emission spectrum is flat enough, i.e., there are no dominant wavelengths; and (iii) lie in a wavelength range that contains the emission wavelength of the high coherent laser used for comparison (638 nm). The last condition is required to have similar imaging specifications (speckle size, resolution, etc.) in all the configurations evaluated. The three laser beams are collimated and aligned in order to propagate along the same path. A 10× telescope is used to expand the laser beams, and the iris (I), with fixed aperture, limits their waist to 3 mm. A cylindrical lens (CL, f = 100 mm) generates a light sheet at its front focal plane, where a galvo mirror (GM) is placed. The light sheet at the GM plane is imaged onto the sample plane by using a telescope composed of a spherical lens (L2, f = 125 mm) and the illumination objective lens (OBJill, 10×, 0.3 NA). The GM scans the beam along the vertical axis of the rear pupil of OBJill, which transforms positions at the pupil to different angle directions at the sample plane. In this way, we generate a pivoting light sheet around OBJill’s working distance (WD), at the centre of the sample plane, as illustrated in Figure 1b. In practice, the numerical aperture of OBJill, NA = 0.3, and the refractive index of the immersion medium, n = 1.33, determine the maximum pivoting angle of the light sheet, $\theta =\arcsin\frac{NA}{n}=$ 13°. In spite of this, to avoid clipping the laser beam at the edges of OBJill’s pupil, we have adjusted the GM for a smaller effective scanning amplitude. Regarding the light sheet dimensions, taking into account the limitations imposed on the beams by I, CL, L2 and OBJill, we have experimentally obtained an illumination profile with thickness, width and height of: 5 μm, 575 μm and 295 μm, respectively, as measured with a fluorescent solution [20]. At the detection side, we use an objective lens (OBJdet) with NA = 0.5 and a 200 mm tube-lens, achieving an effective magnification of 20× on the CCD camera (Hamamatsu Orca R2, 1344 × 1024 pixels, 6.45 µm pixel size). The illumination light sheet is centred and focused at the field-of-view (FOV), defined by the CCD imaged area, for optimum optical sectioning. In addition, we use a set of half-wave plates (HWP) and polarizers mounted on high precision rotation mounts to control the polarization: P1 is an HWP that determines the polarization state at the illumination path, and P2 is a polarizer that serves as a polarization analyser at the detection path. A band-pass filter (BF) with a central wavelength of 641 nm and a bandwidth of 71 nm is placed at the detection side when the LSFM modality is required for fluorescent detection. In such cases, the 514 nm laser is used for red fluorescence excitation. The samples are held in a custom-made immersion chamber (C) using a capillary holder mounted on a 2-axis (*x-z*) motorized stage. For 2D imaging, samples are located using the motorized stage at the centre of the FOV, at the desired axial position. For 3D imaging, samples are sequentially translated in the *x* (axial) direction capturing stacks of optical sections along the desired range.

## 3. Results and Discussion

### 3.1. Polarization Filtering

We first analyse the effect of changing polarization states, vertical or horizontal, at both the illumination (IN) and the detection (OUT) paths of our elastic scattering light sheet microscope using the 638nm laser diode. In this preliminary analysis, we image a tube made of fluorinated ethylene propylene (FEP) filled with 1% agarose. This is a popular sample holder-substrate preparation used in many biological experiments [21,22] and it is an interesting sample to test the possible sources of background and speckle noise in realistic elastic scattering experiments. In our experiments, we focused on the interface between the FEP-tube wall and the agarose.

As in any standard LSFM architecture, in our system, the illumination and the detection paths are mutually orthogonal. This means that the light reaching OBJdet is always the component of the light sheet that is scattered at angles close to 90° with respect to the plane of illumination. These scattered light components are the ones contributing to image formation at the sensor plane.

**Figure 2:**
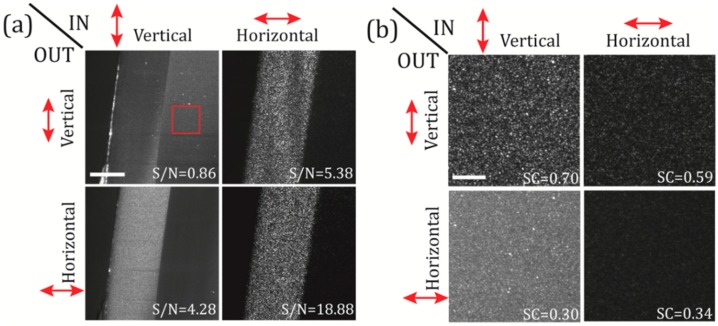
Polarization filtering on a test sample made of a fluorinated ethylene propylene (FEP) tube containing 1% agarose. In (**a**) the FEP-tube wall is imaged changing the polarization state (vertical or horizontal) of the illumination beam (IN), and, selecting the polarization (vertical or horizontal) at the detection path (OUT). The S/N ratio is calculated for the four IN-OUT polarization combinations, as the ratio of the mean intensities of the FEP to the agarose regions. Scale bar 100 μm. In (**b**) the speckle patterns of selected regions-of-interest on the agarose (red square in panel (a)) are shown for the four available polarization configurations. Speckle contrast (SC) is also shown for all configurations; see the main text for details. Scale bar 15 μm. Laser power at the back of OBJill is 6 mW.

In Figure 2a we image a single section of the FEP-agar sample for the four possible combinations of IN/OUT polarizations. In order to quantify the effect of the polarization on the captured images we evaluate the signal-to-noise (S/N) as the ratio of the average intensity of the FEP region (the FEP-tube wall is our test sample) to the average intensity of the agarose region, $S/N={\langle I\rangle }_{sample}/{\langle I\rangle }_{agar}$. Similar to what was previously reported in [12], while in the case of the polarization configuration vertical-IN vertical-OUT the S/N is very poor (0.86), in the polarization configuration horizontal-IN horizontal-OUT the S/N reach much higher values (18.88). This is due to the difference in composition of the two materials analysed. On one hand, the jellified agarose is composed of small particles, smaller than the illumination wavelength, that interact with the incident laser light and produce mostly Rayleigh scattering (RS). When RS is dominant the electromagnetic waves that impinge on the particles in liquid solution, are absorbed and re-radiated with a polarization orientation that depends on the angle *θ* at which those waves get scattered. For *θ* = 90°, corresponding to the angle of the maximal scattered intensity, the scattered light is perpendicular to the plane of oscillation of the molecules and is totally plane polarized. For any other angle, the scattered light is partially plane polarized. Considering that in our microscope’s geometry (see Figure 1) the detection is oriented perpendicular to the illumination direction, in principle, we detect only the light that scatters at the angle *θ* = 90°, i.e., exhibiting a preferential orientation in polarization (vertical). Therefore, the light scattered in the x-direction will tend to be polarized in the y-direction (vertical), and then, the maximum intensity will be detected when the IN polarization is also vertical (and minimum when it is horizontal) [23]. On the other hand, the FEP tube wall is a dense and solid polymeric material composed of large and heterogenic molecules with sizes comparable to or larger than the wavelengths in use. The light scattered by those molecules is better described by the Mie solution to Maxwell’s equations. By trespassing the FEP-tube wall, the light scatters in an anisotropic manner without a clear predictable polarization (depolarization). This explains why the maximum S/N is obtained in the horizontal-IN horizontal-OUT case as the RS from the agarose is minimized. As this polarization configuration is potentially useful to reduce the RS background of the agarose substrate, this also means that any RS originated at the sample of interest will be filtered as well. This will not pose a huge problem as, in general, the structures of interest will have sizes comparable to or larger than the point-spread function (PSF) of the microscope, and objects in such a size range are not likely to scatter light in the Rayleigh regime. In any case, if the RS generated at the sample has to be detected, one can always optimize it by changing the microscope’s polarization configuration. Moreover, it is interesting to note that, although the S/N for the vertical-IN cases are not optimal for scattering detection, this configuration enables the detection of ballistic light reflected at the FEP tube boundaries due to geometric scattering. See the regions of high intensity at the surface of the FEP wall for both vertical-IN cases of Figure 2a.

In addition, to determine in a quantitative manner the effective noise contribution from the agarose scattering we use a parameter known as the speckle contrast (SC), which provides a measure of the speckle pattern development or, in other words, how well defined are the speckle grains [8]. The SC is defined as the ratio of the standard deviation of the intensity fluctuation to the mean intensity of a speckle field, $SC=\sigma_{s}/\langle I\rangle$, and it can take values between 0 and 1. SC values close to the unity imply high visibility of the speckle on the images acquired. In Figure 2b selected regions on the agarose (red square in Figure 2a) are analysed using the SC for the four polarization configurations. In this case, the best speckle noise reduction is obtained for the vertical-IN horizontal OUT and the horizontal-IN horizontal OUT cases, where we get SC values of 0.3 and 0.34, respectively. For instance, it is interesting noting that the minimum SC value were found for the case vertical-IN horizontal OUT (SC = 0.30), although this configuration does not provide the best “visibility” of our test sample. In order to choose the best configuration, one has to take in account also the values of the S/N ratio obtained in Figure 2a. If both S/N and SC are considered, the configuration which maximizes the sample signal and, at the same time, minimizes the RS background from the agarose substrate, is the horizontal-IN horizontal-OUT configuration (S/N = 18.88, SC = 0.34).

### 3.2. Effects of the Temporal and the Spatial Coherence on the Speckle Noise

Now we study how the speckle noise is affected: (i) by reducing the temporal coherence of the light sheet using a SCL instead of a high coherence diode laser, and (ii) by reducing the spatial coherence of the scattering images by using a time-averaged pivoting light sheet. Note that we expect a reduction in the speckle contrast for both cases as both of them rely on the same principle of adding speckle realizations in an incoherent manner. Therefore, the SC can vary from 1 to $1/\sqrt{M}$, where M is the number of independent speckle realizations with equal mean intensities [24].

**Figure 3:**
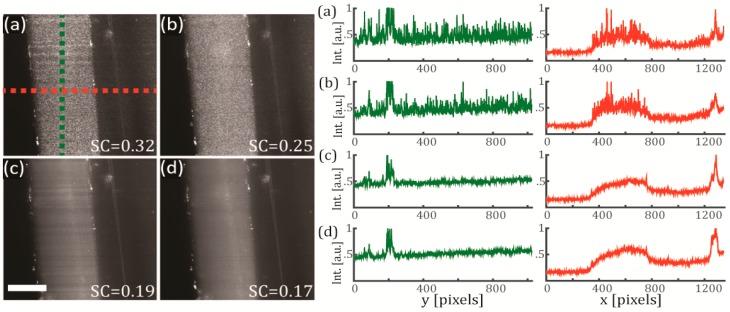
Effects of the coherence reduction on the speckle noise using a sample of FEP and agarose. The test sample is the FEP-tube wall. (**Left**) Single optical sections for the four different cases of study (integration time of 500 ms) and (right) their corresponding line intensity profiles along *y* (green) and *x* (red) axes. In (**a**) a diode laser (high temporal coherence) emitting at 638 nm is used for generating a static illumination light sheet (colored lines are the paths were the line intensity profiles were measured). In (**b**) same configuration as (a), but the light sheet pivots at 100 Hz regarding the centre of the FOV. In (**c**) a SCL (low temporal coherence) emitting a broadband spectrum of 140 nm (centred at 600 nm) is used for generating a static illumination light sheet. In (**d**) same configuration as (c), but the light sheet pivots at 100 Hz regarding the centre of the FOV. Scale bar 100 μm.

In Figure 3 we evaluate the full coherent control of the illuminating light sheet and its effect on the speckle contrast, using the same test sample (FEP-tube wall) as in the previous section. The polarization configuration was set to vertical-IN horizontal-OUT, and the camera integration time was set to 500 ms. In Figure 3a a diode laser with high temporal coherence was used as an illumination source for creating a static light sheet. As seen from the figure, the speckle noise is clearly present on the obtained image. In Figure 3b we use the same laser but this time the light sheet is pivoting at 100 Hz, obtaining a reduction of the SC from 0.32 to 0.25. In Figure 3c a SCL with low temporal coherence is used to create a static illumination sheet, reducing the speckle noise down to SC = 0.19. In Figure 3d we use the same SCL light source but this time the light sheet is pivoting at 100 Hz, obtaining a further reduction of the SC from 0.19 to 0.17. Therefore, we can conclude that using a configuration that combines the use of a SCL with a pivoting approach is an effective way of reducing the SC. In our test sample, the SC was reduced by almost a half by changing from the diode-laser non-pivoting (high coherence) configuration to the SCL-pivoting (low coherence) configuration. These observations are also confirmed by comparing the line intensity cross-sections of the four configurations studied, as shown in Figure 3 (right). The intensities are plotted along the green and red dashed lines defined on Figure 3a. While the profiles corresponding to cross-sections from panels (a) and (b) exhibit strong fluctuations and discontinuities, the profiles from panels (c) and (d) are much smoother and disclose spatial information that otherwise would be hidden within the speckle noise.

These results clearly demonstrate that by implementing a combined control of the coherence in an elastic scattering light sheet microscope, it is possible to reduce the speckle noise and recover the intrinsic contrast of the sample in the captured images. An additional benefit of using the pivoting light sheet approach is that it helps to mitigate other types of image artifacts such as the horizontal shadowing typical of the light sheet geometry. Notice that some horizontal shadows are quite visible in both Figure 3a,c but not so much in their counterparts captured with a pivoting light sheet, Figure 3b,d.

### 3.3. Results Obtained by Using Biological Samples

To show the full potential of our enhanced elastic scattering light sheet microscope, we have tested both polarization filtering and coherence control on relevant biological samples.

Multicellular tumour spheroids (MCTS) are aggregates of cells with nearly spherical shape which serve as a biological model for studying the growth dynamics of cancerous tumours [25]. Imaging inside large spheroids is challenging as MCTS can grow up to diameters of several hundred of micrometers inducing aberrations and scattering on the light propagating inside them. In this sense, LSFM has become the tool of choice for imaging MCTS due to its versatility for imaging 3D volumetric samples [26,27,28].

**Figure 4:**
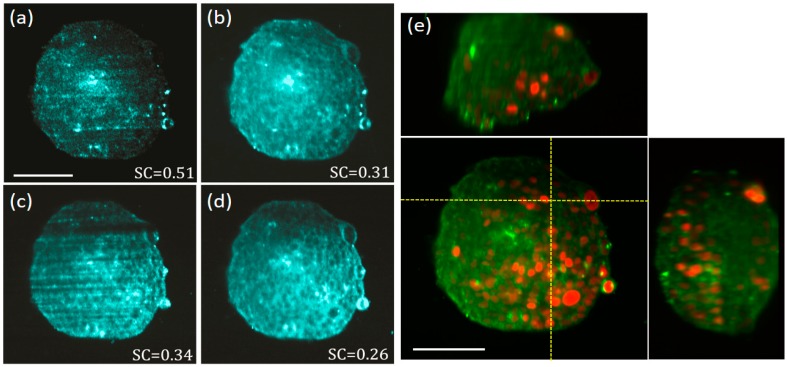
Elastic scattering light sheet imaging of a multicellular tumour spheroids (MCTS) with fluorescently labelled nuclei. Polarization was set to horizontal-IN horizontal-OUT configuration. Elastic scattering images of a single optical section of the MCTS obtained by using: (**a**) a diode laser (high temporal coherence) emitting at 638 nm and a static illumination light sheet, (**b**) the same diode laser of (a), but with the light sheet pivoting at 100 Hz with respect to the centre of the FOV, (**c**) a SCL (low temporal coherence) emitting a broadband spectrum of 140 nm (centred at 600 nm) and a static illumination light sheet, and, (**d**) a SCL as in (b) but the with light sheet pivoting at 100 Hz with respect to the centre of the FOV. (**e**) 3D orthogonal views (centre panel front-view, top panel top-view, right panel side-view), of the same MCTS sample where the fluorescent nuclei (red) and the elastic scattering data (green), obtained with the SCL and the pivoting light sheet, are merged together for comparison. The MCTS nuclei are labelled with the mCherry fluorescent protein. Laser powers at the back of OBJill are 3 mW and 1 mW for elastic scattering and fluorescence imaging modalities, respectively. Scale bars 100 μm.

We have tested our enhanced light sheet elastic scattering microscope on a fixed 200 μm diameter MCTS expressing the mCherry fluorescent protein in the nuclei. The MCTS was mounted inside a piece of FEP tubing filled with 1% agarose. In Figure 4a–d we show the results obtained for a single optical section of the MCTS obtained with our elastic scattering light sheet microscope. In this case, the polarization was set to horizontal-IN horizontal-OUT configuration to minimize the scattering background generated by the agarose. In Figure 4a a diode laser with high temporal coherence is used as illumination source for creating a static light sheet. As expected, speckle is evident on the captured MCTS image, and the internal structures are not clearly visible due to the strength of the speckle noise (SC = 0.51).

In Figure 4b we use the same laser but this time the light sheet is pivoting at 100 Hz, obtaining an important reduction of the SC from 0.51 to 0.31. In Figure 4c a SCL with low temporal coherence is used to create a static illumination sheet. In this case, speckle noise contrast is reduced down to 0.34 as compared with the image obtained with the high coherence laser. Nevertheless, in this configuration, the image quality is degraded by the presence of the light-sheet specific shadowing artifact that dominates the contrast of the image. In Figure 4d we use the same SCL but this time the light sheet is pivoting at 100 Hz. Using this method, we have obtained a further reduction of the SC from 0.34 to 0.26. Thanks to this, from Figure 4d we are now able to distinguish individual cell locations on the MCTS, revealed as dark round regions, avoiding the disturbance of speckle and other artifacts. Although the exact spatial characteristics of light scattering produced by individual cells, and cell aggregates, is quite complex [29,30], in this case, the dominant scattering signal seem to be originated at the cells surrounding cytoplasm (but not at the nuclei). This is confirmed by the data of Figure 4e where 3D orthogonal views of the enhanced elastic scattering data (green) is merged with the fluorescence signal (red) obtained with the LSFM modality of our microscope. As we are certain the fluorescent signal detected is generated at the cell nuclei of the MCTS, it is clear from the merged data that elastic scattering and fluorescence signals inversely correlate. Thus, we conclude that the elastic scattering signal for the MCTS sample comes from the biological material surrounding the nuclei, confirming the complementary nature of both sources of contrast. Therefore, light sheet elastic scattering offers relevant information on the structure of the MCTS and could be used to quantitatively measure the proliferation of the cells inside the MCTS, or as an alternative to membrane/cytosolic staining. Another interesting effect that can be observed in both frontal and top views of the Figure 4e is the degradation of the light sheet upon propagation inside the MCTS (light sheet propagates from right to left in the front view). A close observation reveals that these images become progressively blurry towards their left side, revealing a compromised optical sectioning at those regions. It is well known that MCTSs are very challenging samples for all kinds of optical imaging, as they are large and dense aggregates of heterogeneous cellular material. In particular for LSFM imaging, it has been shown that in most of the cases, when visible light is used for excitation and detection, the limited light penetration depth reduces the volume of clear imaging to a region around the quadrant of the MCTS facing OBJdet and closer to the incidence side of the light sheet [28]. As for our enhanced elastic scattering light sheet microscope, we used visible light from the SCL, and the captured 3D images of Figure 4 are also affected by this limited penetration.

Finally, we have tested our scattering-based light sheet microscope on an equally challenging biological sample, a 48 hpf fixed zebrafish (ZF) embryo. ZF is an important model organism vastly used for research in numerous biological disciplines. Due to its outstanding performance for imaging living specimens, LSFM has become the instrument of choice for research on developmental biology using ZF models [31]. In this work, we evaluate the ability of our light sheet elastic- scattering microscope of performing full 3D speckle-free imaging on a ZF sample.

**Figure 5:**
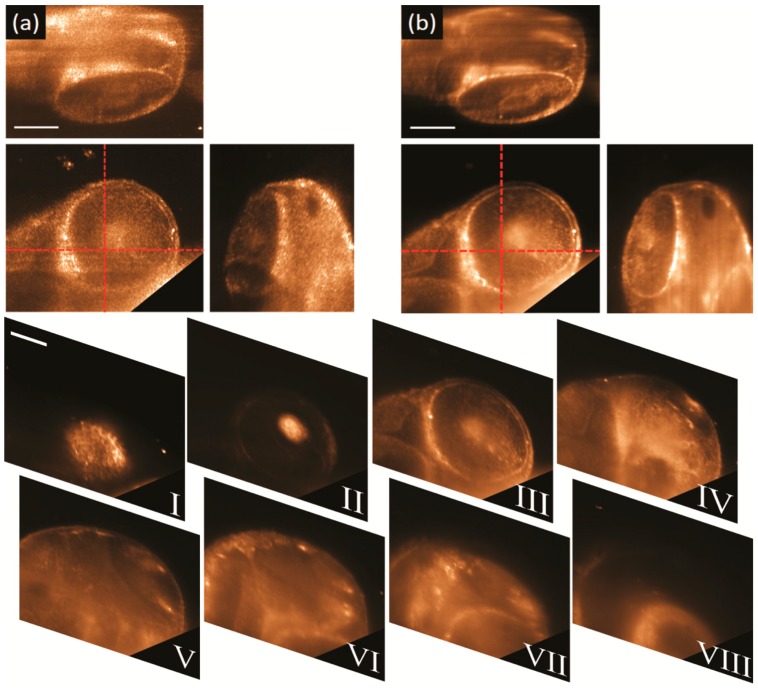
3D elastic scattering light sheet microscopy of the head of a 48 hpf zebrafish embryo. Polarization was set to horizontal-IN horizontal-OUT configuration. 3D orthogonal views (center panel front-view, top panel top-view, right panel side-view) of the data taken with: (**a**) a high coherence (638 nm) diode laser and a static illumination light sheet, and, (**b**) a low temporal coherence SCL emitting a broadband spectrum of 140 nm (centred at 600 nm) and with the light sheet pivoting at 100 Hz with respect to the centre of the FOV. Corner cut in the front views were made to remove the intense scattering signal coming from the embryo’s yolk. From panels I to VIII, stack of eight consecutive sections separated each other 40 μm. Laser power at the back of OBJill is 1 mW for both datasets. Scale bars 100 μm.

Figure 5 shows the captured 3D elastic scattering light sheet microscopy data of a fixed 48 hpf embryo. The embryo was mounted inside a piece FEP tubing filled with 1% agarose. The head region around embryo’s eye was selected for imaging, as it contains some strong and recognizable features such as the eye lens and the retina surface [32,33]. Figure 5a shows the 3D orthogonal views of the captured data using a static light sheet generated with a high coherence (638 nm) diode laser. From the top and lateral views, it is clear that full optical sectioning is achieved by using a light sheet illumination, as only a little amount of out-of-focus light is seen in the views but deep inside the embryo.

In contrast, it is evident from the same data that the imaging quality is affected by the speckle noise as it prevents the clear observation of the characteristic features of the eye. Figure 5b shows the 3D orthogonal views of the captured data using the SCL (low temporal coherence) with the light sheet pivoting at 100 Hz with respect to the centre of the FOV. The 3D views reveal that by using both temporal and spatial coherence control it is possible to cancel out the speckle noise. By using a pivoting, low temporal coherence light sheet, the retinal pigmented epithelium (RPE) (bright circumference in the front view) can be imaged using the scattered light and without any fluorescent labelling. Moreover, some other finer cellular structures, that resemble retinal neurons, are now evident in front and top views of Figure 5b, without any interference of speckle or shadow artifacts. Finally, in order to corroborate the optical sectioning capability of our elastic-scattering light-sheet microscope a stack eight consecutive sections, acquired along a total distance of 280 μm, are shown in Figure 5I–VIII. It is evident from the images that the light sheet confinement provides clean optical sectioning and minimal cross talk between consecutive sections.

All these results show that our technique is capable of non-invasive pristine structural imaging that would be useful for non-specific volumetric inspection of the intrinsic architecture of all sorts of biological samples.

## 4. Conclusions

In conclusion, elastic scattering light sheet microscopy is a novel light sheet imaging modality suitable for label-free structural imaging. In order to enhance the imaging quality in this configuration we have proposed to implement: (i) polarization control, which enables contrast selectivity and cancelling substrate background; and, (ii) temporal and spatial coherence reduction, which enables extracting the endogenous intrinsic contrast from the speckle noise. Implemented in this manner, elastic scattering light sheet imaging provides useful complementary structural information to standard LSFM experiments, as shown for MCTS samples. Additionally, in a more general context, it has the potential to provide relevant morphologic details of the samples, similar to histological sections, but in a non-destructive way, as shown for the ZF embryo sample. Finally, elastic scattering light sheet microscopy is a promising technique which could enable new and interesting experiments, for example, as an alternative to LSFM in applications that are limited by low SNR, such as functional imaging or fast volumetric structural imaging [34].
